# Genomic Variations and Mutational Events Associated with Plant–Pathogen Interactions

**DOI:** 10.3390/biology11030421

**Published:** 2022-03-10

**Authors:** Aria Dolatabadian, Wannakuwattewaduge Gerard Dilantha Fernando

**Affiliations:** Department of Plant Science, Faculty of Agricultural and Food Sciences, University of Manitoba, Winnipeg, MB R3T 2N2, Canada; dilantha.fernando@umanitoba.ca

**Keywords:** genomic variation, mutational events, breeding for resistance, plant–pathogen interactions

## Abstract

**Simple Summary:**

Plants, unlike animals, do not have defender cells or an adaptive immune system. Instead, plants rely on each cell’s innate immunity and systemic signals emitted from infection sites. On the other hand, not all plants, even within the same species, are genetically identical, and their genetic backgrounds determine how well they respond to stress factors. Through evolution, plants have acquired various defense mechanisms that play important roles in the never-ending fight between plants and pathogens. Genetic variation in relation to plant disease resistance can thus be contextualized to provide new insights into these defense mechanisms and evolutionary processes that lead to resistance to pathogens. By focusing on genetic variations and mutational events linked with plant–pathogen interactions, the paper explores how genome compartments facilitate plant and pathogen evolutionary processes.

**Abstract:**

Phytopathologists are actively researching the molecular basis of plant–pathogen interactions. The mechanisms of responses to pathogens have been studied extensively in model crop plant species and natural populations. Today, with the rapid expansion of genomic technologies such as DNA sequencing, transcriptomics, proteomics, and metabolomics, as well as the development of new methods and protocols, data analysis, and bioinformatics, it is now possible to assess the role of genetic variation in plant–microbe interactions and to understand the underlying molecular mechanisms of plant defense and microbe pathogenicity with ever-greater resolution and accuracy. Genetic variation is an important force in evolution that enables organisms to survive in stressful environments. Moreover, understanding the role of genetic variation and mutational events is essential for crop breeders to produce improved cultivars. This review focuses on genetic variations and mutational events associated with plant–pathogen interactions and discusses how these genome compartments enhance plants’ and pathogens’ evolutionary processes.

## 1. Introduction

Plant diseases caused by bacteria, fungi, viruses, nematodes, and protists have occurred throughout the history of plant colonization on Earth. As a result of plants’ continued interactions with pathogens, plant genomes have been shaped through coevolution processes, with pathogen-imposed selection pressures leading to selection signatures in the genome [1,2]. Nonetheless, the effects of pathogens vary from minor symptoms to severe attacks in which large-scale planted areas are destroyed, such as the jarrah (*Eucalyptus marginata*) dieback disease caused by *Phytophthora cinnamomi* in Western Australia [3]. Plant pathogen populations vary in time, space, and genotype and can evolve and overcome the resistance that plant breeders incorporate into new varieties, especially when major genes are involved. Nevertheless, genetic resistance is still a feasible option for controlling plant diseases. Thus, there has been a boom in molecular breeding research to uncover genetic resistance in recent years. In today’s genomic era, plant disease resistance researchers employ genotyping by sequencing and high-throughput phenotyping methods to identify, map, and track resistance genes. In addition, the development of gene-editing technologies, including CRISPR/Cas, TALENs, and zinc finger nucleases (ZFNs), has provided promising opportunities to create genetic diversity for resistance breeding. Due to its extraordinary efficiency, relative simplicity, and low risk of off-target effects, CRISPR/Cas9 provides the best strategy for genome editing [4,5]. These techniques have developed our knowledge of the complicated interactions between plants and pathogens enabling the discovery of fundamental aspects in susceptible and resistant interfaces. The current review first summarizes different types of plant responses modulated by plant interactions with pathogens, then describes genetic variations and mutational events. We also analyze previous studies that clarified the role of these events in interactions between plants and pathogens. These studies shed light on the molecular basis of host defenses at various levels in resistant and susceptible interactions.

## 2. Plant–Pathogen Interactions

### 2.1. Gene-for-Gene Relationship 

Plant disease control has historically relied on traditional breeding for disease resistance. It was not until the 1940s that Harold Henry Flor published his important study on the interaction between flax and its obligatory rust pathogen, *Melampsora lini* [6], resulting in the formulation of the gene-for-gene hypothesis, wherein a plant harboring a resistance gene resists pathogens that contain complementary avirulence (*Avr*) genes [7,8]. *Avr* genes encode small, secreted proteins called AVR proteins or effectors that are recognized by cytoplasmic R proteins inside the host cell. *Avr* genes are pathogen genes that only encode a conditionally recognized protein by plants with the corresponding *R* gene. However, even if the plant contains an *R* gene, the pathogen may still cause disease, even though the pathogen possesses the avirulence gene. Rapid breakthroughs in ‘omics’ technologies have accelerated the identification of *Avr* genes in plant pathogenic fungus. To date, many *Avrs* have been cloned from the filamentous fungi that infect a wide range of agriculturally important crops [8]. For example, the gene-for-gene relationship between *Leptosphaeria maculans* and *Brassica napus* has been widely investigated. In total, 23 resistance genes and 14 avirulence genes have been identified, of which three *R* genes and eight *Avr* genes have been cloned. Recently, Neik et al. [9] reported the cloning of *AvrLmS* and *AvrLep2* and found that these *Avr* genes, which were previously described as different avirulence genes, to be perceived by different resistance genes; *RlmS* and *LepR2*, were found to be the same. Additionally, *Rlm4* and *Rlm7*, which confer resistance to *L. maculans*, were found to be alleles of the *Rlm9* wall-associated kinase-like resistance locus [10]. 

This gene-for-gene plant disease resistance is linked to another response called hypersensitive response (HR) [11]. HR gene expression is triggered when an incompatible pathogen infects resistant plants. It is described by localized cell death at the site of infection, forming a physical barrier that limits the pathogen’s access to nutrients and prevents the pathogen from spreading to uninfected tissues [12]. The most common HRs are those caused by fungi, oomycetes, bacteria, and viruses, although HRs can also be caused by nematodes [13] and insects [14]. HR is more effective against biotrophic pathogens than necrotrophic pathogens, which need dead tissue to complete their life cycle. In the case of hemibiotrophic pathogens, in which the initial interaction is biotrophic and then switches to necrotrophic, HR may benefit the host during early, but not later, stages of infection [15,16]. 

### 2.2. Zigzag Model of Plant–Pathogen Interactions

**Figure 1:**
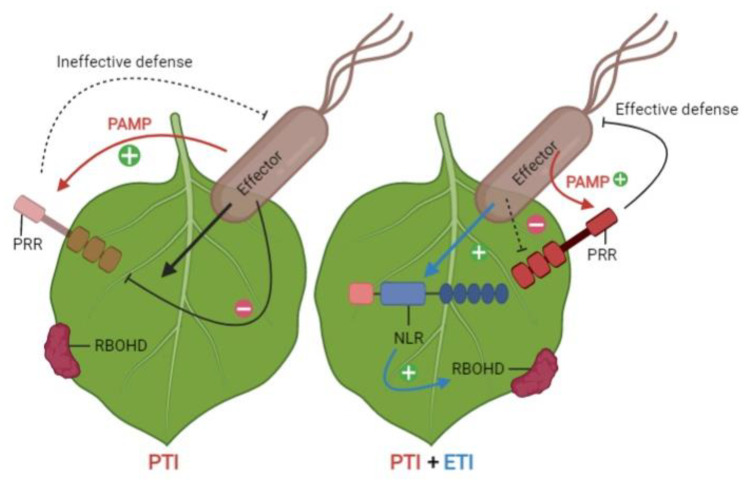
A two-tiered immune system consisting of pattern-triggered immunity (PTI) and effector-triggered immunity (ETI) to cope with pathogen attack. PAMP: Pathogen associated molecular patterns, PRR: Pattern recognition receptors, NLR: Nucleotide-binding site-leucine-rich repeat, RBOHD: NADPH oxidases belong to the respiratory burst oxidase homolog.

According to the zigzag model of plant–pathogen interactions, induced defense consists of two layers. The first layer is called microbe- or pathogen-associated molecular-pattern (MAMP or PAMP)-triggered immunity (PTI) [17,18]. Pathogens have developed a wide range of effectors—small molecules, mainly proteins encoded by *Avr* genes—to control host cellular processes and form parasitic relationships [19,20]. These components are recognized by plant receptors, a related class of proteins known as pattern recognition receptors (PRRs), which initiate PTI. To avoid PTI, pathogens deliver effector proteins inside host cells, interfering with defense responses. Plants perceive effectors through resistance (*R*) genes and activate a more robust and faster defense response, known as effector-triggered immunity (ETI) [21] (Figure 1). The mutual potentiation of immunity by PTI and ETI components is required to defend against host-adapted microbial infections successfully. However, when pathogen effectors suppress PTI, pathogens can successfully infect susceptible hosts; in the absence of effective R proteins, ETI can be overcome, eventually leading to effector-triggered susceptibility (ETS) [22,23]. 

Two recent studies investigated the relationship and interactions between PTI and ETI using complementary approaches in *Arabidopsis* [24,25]. These studies revealed that both defense layers are necessary for mounting a strong defense response, as PTI and ETI complement each other. ETI potentiates the PTI pathway, which is essential for complete resistance, by increasing the number of signaling components. On the other hand, PTI increases ETI’s defense output by magnifying the hypersensitive response. These findings suggest a novel model of plant immunity, in which all the components must be completely functional rather than working on two mostly separate levels [26]. 

### 2.3. Systemic Acquired Response and Induced Systemic Resistance 

Other immunological responses in plants include the systemic acquired response (SAR) [27,28]. SAR is triggered at an infection site and prevents disease from spreading from infected to healthy tissues by activating and expressing pathogenesis-related proteins [29,30]. It was suggested that the immunological memory of SAR can be passed down from generation to generation through trans-generational immunological memory [30]. For example, when *Arabidopsis* plants were inoculated with *Pseudomonas syringae*, salicylic acid (SA) accumulation rapidly increased, and signaling pathway transcripts with boosted disease resistance were observed in the plants’ next generation, suggesting that plants can pass on resistance to subsequent generations [31,32]. Induced systemic resistance (ISR) is a resistance mechanism in plants triggered by infection [33]. Unlike SAR, which is induced by pathogens and insects in systemic tissues of plants, ISR is mediated by beneficial microbes such as bacteria and fungi in the aerial tissues of plants [28,33]. For example, plant growth-promoting rhizobacteria (PGPR) suppress diseases via antagonism between the bacteria and soil-borne pathogens, as well as by inducing systemic resistance in the plant against both root and foliar pathogens [34]. PGPRs provoke ISR via elicitors. Among these elicitors, there are MAMPs (such as flagellin, chitin, and lipopolysaccharides) [35,36] and volatile organic compounds or siderophores [36,37]. MAMPs are PRRs perceived by PRRs, while other elicitors can be perceived by other receptors [36,38,39]. These elicitors, upon perception, trigger the ISR through the action of various plant hormones [38,39]. Additionally, elicitors can cause drastic changes in plant growth patterns, generally by altering hormone signaling [38,39]. Among the hormones implicated in the ISR, jasmonic acid (JA), ethylene (ET), and auxin, play key roles [40,41]. PGPRs activate the SA-dependent SAR pathway by producing SA at the root surface, whereas other rhizobacteria trigger different signaling pathways independent of SA. The existence of an SA-independent ISR pathway was studied in *Arabidopsis thaliana*, which is dependent on jasmonic acid (JA) and ethylene signaling [34]. The complexity and diversity of the signal pathways involved in ISR were highlighted by the activation of both the SA and JA/ET signaling pathways in ISR caused by beneficial microbes [42].

### 2.4. Recognition Models

**Figure 2:**
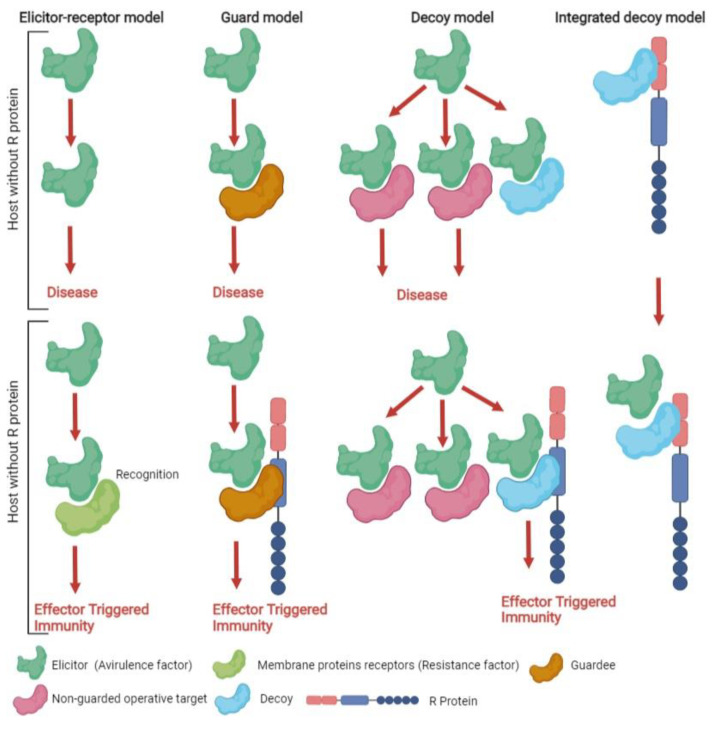
Comparison of four proposed models of AVR recognition by R proteins.

Resistance proteins recognize AVR through four different coexisting models (Figure 2). (1) In the elicitor–receptor model, the AVR protein is directly recognized by its corresponding R protein to initiate defense responses [43]. *Avr* gene products are very small and colocalized with *R* gene products, reinforcing this ligand-receptor hypothesis. (2) In the guard model, AVR and R proteins indirectly interact. The R protein recognizes changes in the host target protein of an effector, known as a “guardee” [44]. The most convincing evidence for the guard hypothesis was found in *A. thaliana* bacterial *R-Avr* systems [45]. The guard model can be compared with an altered guard model in which the effector targets several plant proteins. (3) In the decoy model, the R protein traps the AVR protein by detecting changes in a protein called a “decoy” that resembles the effector target [46]. (4) In the integrated decoy model, non-canonical domains that imitate the effector target are included in the NLRs and serve as “decoys” [46].

## 3. Genomic Variation and Mutational Events in Hosts and Pathogens

Genomic variation describes the differences between individuals’ genomes. More precisely, genomic variation is a DNA segment that differs in length, orientation, copy number, or chromosomal location between different individuals [47]. Genomic variation encompasses various microscopic (visible under a microscope—for example, chromosomal rearrangements) and submicroscopic (>1000 bp) types of variation in a species’ genome, resulting in deletion; duplication; changes in sequence, such as a single nucleotide polymorphism (SNP); and the creation of new genes, resulting in heritable phenotypic changes seen within and between species [25]. Genomic variations play a significant role in plant adaptive evolution, functional diversity, and phenotypic diversity [48]. 

There are several causes of genetic variation such as mutation and genetic recombination [49]. Genomic structures and mutational events that allow rapid evolution include AT-rich isochores, length polymorphism and chromosomal polysomy, chromosomal rearrangements, conditionally dispensable chromosomes, copy number variation (CNV), de novo genes, epigenetic modification of gene expression, horizontal gene/chromosome transfer (HGT/HCT), hybridization, insertions/deletions (indels), polyploidization, repeat-induced point mutation (RIP), RIP leakage, single nucleotide polymorphisms (SNPs), and transposable elements (TEs). In the following, we present some significant genomic features and mutational events that have known functions in plant–pathogen interactions and evolution. 

### 3.1. Transposable Elements 

Transposable element (TE) insertions and deletions, originally considered selfish DNA or ‘genome parasites’ [50], are mobile genetic components that can jump across genomes. Transposition events are among the most common genetic variations in plants that can result in gene activation, inactivation, duplication, and even the appearance of a new gene [51]. TE insertion can disrupt the open reading frame (ORF) by invading the space inhabited by protein-coding genes and yield abnormal phenotypes [52]. In fungal phytopathogens, TEs play an important role in rapid evolution by affecting genome plasticity [53,54], pathogenicity [55], host range [56], and evolution [57,58]. In some fungal plant pathogens, genome compartments on core chromosomes act as accessory islands and encode virulence determinants [59]. In *L. maculans* ‘brassicae’ and *Zymoseptoria tritici*, TE-rich genome sections are exemplified by epigenetic alterations that are further associated with diverse patterns of transcription and accumulation of mutations [60]. These compartments can be produced by structural changes or develop in regions with suppressed recombination [61]. For example, in *Verticillium dahliae* and *Z. tritic*, accessory genome sections originate through structural changes and unfaithful DNA repair across repeated sequences [59]. Pathogen genomes with low TE can still have fast-developing genomic regions that promote effector evolution. 

The activity of transposable elements plays a significant role in effector gene evolution [59,62]. For example, although *Ustilago maydis*, *Sporisorium scitamineum,* and *S. reilianum* have low TE content, the TEs are remarkably linked to virulence gene clusters [63]. The association between TEs and effector genes indicates that elevated mutation levels in repetitive genome sections support effector improvement and adaptation, as shown in *Magnaporthe oryzae* and *Fusarium oxysporum* [62,64,65]. As demonstrated in *M. oryzae*, TEs are frequently found near pathogenicity factors [66]. The TE-pathogenicity gene involvement was also demonstrated in other fungal pathogens—for example, *Mycosphaerella fijiensis*, which causes black Sigatoka in bananas [67] and *M. graminicola,* which causes *Z. tritici* blotch in wheat [68]. TE insertion may alter a fungal pathogen’s pathogenicity and host specificity by generating genetic variations in virulence factors to evade detection by the host plants. Collectively, the presence and actions of TEs promote variability and adaptability.

### 3.2. Repeat-Induced Point Mutation 

The repeat-induced point (RIP) mutation is a genome defense mechanism specifically found in fungi that protects against the harmful effects of repetitive genomic regions and TEs by mutating cytosine to thymine in repetitive sequences [69]. The RIP pathway protects the fungal genome from the genetic implications of repeated sequence elements, so-called “selfish” sequences, especially those connected with transposable elements [69,70]. The spread of duplicated sequences into neighboring nonrepetitive regions is called RIP leakage [51]. RIP was first identified in *Neurospora crassa* [71]. RIP-like C: G to T: A transitions were reported in the sequences of transposable elements in several fungi such as *Aspergillus fumigatus* [72], *Aspergillus nidulans* [73], *F. oxysporum* [74], and *Magnaporthe grisea* [75]. RIPs are prevalent in *L. maculans* [76], as shown by the degeneration of the retrotransposons (found in the *AvrLm1-AvrLm6* regions), as well as the low GC content in corresponding retrotransposon-rich isochores. Furthermore, in *L. maculans* ‘brassicae’, the RIP mutation can play a crucial role in transposable element silencing and effector evolution [62]. Furthermore, it was shown that RIP operates in *M. grisea* during the sexual phase [77]. The development of specific genes is also influenced by the emergence of RIP-driven lineage-specific regions [62]. The widespread conservation of RIP indicates that RIP is mostly useful for fungal survival and plays critical roles in genome development and evolution, supporting or hindering gene variety and the revolution of novel genes [78].

### 3.3. AT-Rich Isochores

The AT-rich isochore is a region with high content of thymine and adenine residues. AT-rich isochores usually concur with deactivated repetitive elements [51]. AT-rich regions can arise through a variety of mechanisms such as repeat-induced point mutation (RIP), a fungal-specific process mainly considered a means of preventing transposon propagation [69,79]. In most fungi, AT-rich regions are a hallmark of RIP that aim for repetitive DNA and reduce GC-content [79]. The AT-rich region is where DNA synthesis is initiated and the replication complex is formed. High AT-content causes lower thermodynamic stability, which describes the role of AT in the initiating of the replication process [80]. In fungal genomes with substantial numbers of AT-rich regions, a bimodal pattern of GC-content bias can be observed. The *L. maculans* genome was the first fungal genome published with a considerable proportion of AT-rich regions (~33% of the assembly) [62]. Since then, AT-rich regions have been discovered in various fungal genomes such as *Passalora fulva* [81], *Blastomyces dermatitidis* [82], multiple *Epichloë* spp. [83], and *Z. tritici* [84]. Studies on genes encoding avirulence/effector-like proteins such as *L. maculans* genes *AvrLm6*, *AvrLm4-7,* and *AvrLm1,* have increased interest in AT-rich regions [85]. In *L. maculans*, it was reported that like all other *AvrLm* genes, *AvrLmS-AvrLep2* exist in an AT-rich genome environment; encode for small, secreted proteins rich in cysteines; and are extremely overexpressed in the initial cotyledon infections [9]. In *Venturia inaequalis*, the region comprising *AvrVg* is located in isochores with significantly different GC content [86]. This organization is also recognizable in the genomes of *M. fijiensis* and *Passalora fulvum*, which have effector-encoding genes in repeat-rich regions [81]. In a study on *Lupinus angustifolius* L., 22 genes were linked with AT-rich regions. While none were expected to be effector candidates, four continued the Pfam-related domain [87]. AT-rich regions were examined in *Pyrenochaeta* *lycopersici* ER1211 and *L. maculans* genomes in another work. AT-rich regions made up about one-third of the *L*. *maculans* genome and ~10% of the *P*. *lycopersici* ER1211 genome [79]. It was suggested that pathogenic fungi with putative effector genes located near AT-rich regions have competitive evolutionary power [88]. 

### 3.4. Chromosomal Rearrangements and Homeologous Exchanges

A chromosomal rearrangement encompasses different events, including duplications, inversions, and translocations of pieces of chromosomes between the sub-genomes. Sequence exchanges between homeologous chromosomes in polyploid plants result in immediate gene deletions and amplification or homeologous exchanges (HEs) [48,89]. HEs are caused by chromosome mispairing between two genomes that are ancestrally linked. Increased homoeologous exchanges (HEs) and gene conversion events result from a meiotic chromosomal pairing between homoeologous chromosomes with a high degree of sequence identity [90]. It was shown that HEs generate novel gene combinations and phenotypes in a range of polyploid species [91,92]. For instance, gene deletions and HEs between sub-genomes in *B. napus* were shown to reduce seed glucosinolate content [93]. The structures of plant pathogens genes simplify the rapid rearrangements and genomic variation in virulence-associated regions [94]. These rearrangements include chromosomal length variations on a broad scale and the presence of isolate-specific supernumerary chromosomes (small and non-essential chromosomes in addition to the standard chromosomes) [95]. In eukaryotic pathogens, supernumerary chromosomes can be observed at different rates [94,96]. Supernumerary chromosomes have been linked to establishing novel virulence features in several fungus species [64]. The homologous exchange was defined by Shi et al. [97] as an alternate mechanism by which CNV-associated disease resistance QTLs evolved. Quantitative disease resistance was previously linked to homoeologous recombination [98] and the presence/absence of variation [99] in *B. napus*. In addition, Song et al. [100] discovered the genetic diversity affecting disease resistance to be enhanced in genomic regions affected by structural variation, including that caused by homoeologous recombination [101]. Several publications discuss how the genetic rearrangement between fungal isolates contributes to pathogenesis, whether by parasexual recombination, sexual recombination, or hybridization [102,103].

### 3.5. Presence/Absence Variation

Insertions/deletions (InDels) are small fragments of DNA (a few nucleotides up to 50 bp) that are present or absent compared to a reference genome. InDels are prevalent in many species and cause frame shifts by deleting or altering genes [51]. In contrast, presence/absence variation (PAV) is found in the size ranges of genes (up to a few kb) and result in severe functional and phenotypic changes [99]. Homeologous exchanges have also been the primary cause of gene PAV [91]. Since discovering PAV in the *RPM1* gene in *Arabidopsis* [104], many PAVs have been found in disease resistance genes in different species [105,106,107,108]. It was reported that PAV is a key determining factor of *Verticillium longisporum* resistance such that both short- and long-range PAV assist with *V. longisporum* resistance in canola [99]. Gabur et al. [99] also stated that PAVs in the genes primarily implicated in cell wall integrity, growth, and alteration are colocalizing with major resistance QTL in a *B. napus* population. In addition, Bakker et al. [109] showed that the concentrations of cell wall-associated components are considerably associated with *V. longisporum* resistance. In *L. maculans, V. dahliae, Phytophthora infestans, Z. tritici,* and *M. oryzae*, many effector genes show within-species PAV and remarkable connections with transposable-element-rich regions of chromosomes [57,59,110,111]. Despite these findings, little is known about the extent of gene PAV in fungal plant pathogens [112]. One reason for this paucity of data is that a pathogen’s virulence is usually a quantitative trait [113], suggesting that the PAVs of effector genes may be a less common mechanism of coevolution than that in crops, in which virulence is more often a binary trait, with resistant varieties completely preventing infection [96]. Additional functional characterization of PAV genes may help enhance our perception of disease resistance mechanisms and develop resistance via manipulation for future plant breeding programs.

### 3.6. Copy Number Variations

Copy number variations (CNVs) are chromosome insertions, deletions, and/or duplications, and are generally described as a DNA fragment with a different copy number than the reference genome [114]. CNVs implicate DNA segments usually larger than 1 kb in length [115]. CNVs can be inherited from a previous generation or emerge de novo because of duplication/deletion. The fixation of CNVs by drift or selection may contribute to genetic novelty, leading to species adaptations to stressful or new environments [116]. The biological roles of CNVs range from an apparent lack of influence on the overall variability of physiological features through morphological variability to, altered metabolic states, susceptibility to infectious diseases, and interactions between hosts and microbes. As a result, CNVs have great potential to contribute to population diversity [117]. Copy number variations affect many traits, including an organism’s fitness and disease susceptibility, and contribute to co-evolutionary processes between pathogens and hosts or symbionts [118]. Plant disease defense genes were shown to have CNV in various species [107,119,120,121,122,123]. For instance, *Rhg1* confers resistance to soybean cyst nematodes and seems to act via the multiplication of the locus [121]. In a previous study, Qutob et al. [124] identified *Avr1a* and *Avr3a* from *P.* *sojae* and showed how the copy number variation and transcriptional differences of these *Avr* genes represent mechanisms for the evasion of *Rps*-mediated immunity. It was reported that *R* genes present higher CNVs than the rest of the genome [125]. For example, high levels of CNV were found in maize (129 *R* genes) and rice (508 *R* genes) [126]. 

CNVs were found in various plant pathogens, especially fungi, with some promising instances in an express link between CNVs and pathogenicity. For instance, grape powdery mildew (*Erysiphe necator*) can be controlled by sterol demethylase inhibitor (DMI) fungicides. A point mutation in the target gene *EnCYP51*A is a known mode of resistance to DMIs; however, resequencing DMI-resistant *E. necator* isolates showed frequent increases in the copy number of the mutant allele [127]. The authors discovered a link between a higher *EnCYP51* copy number and enhanced gene expression.

### 3.7. Single Nucleotide Polymorphisms 

The replacement of a single nucleotide at a specific position in the genome is called a single nucleotide polymorphism (SNP). SNPs can occur within coding regions in amino acid substitutions, mis-splicing, or premature stop codons. SNPs have a broad distribution and can be detected in any region of a gene, mRNA, or intergenic region [48]. SNPs can result from deficiencies in DNA polymerase replication during meiosis/mitosis or damaged DNA [51]. With the advent of high-throughput genotyping technologies, genome-wide association or multi-SNP association approaches were developed as helpful tools for analyzing the interactions of complicated genetic characteristics in plants, including disease resistance [128]. Genetic variation can be assessed using phenotypic data in plant and pathogen species, and genome-wide association studies (GWAS) can be used to find genes and link them to phenotypes [129]. SNP discovery using GWAS analysis is feasible through various target-enrichment or reduction-of-genome-complexity methods such as genotyping-by-sequencing (GBS) [130] and the restriction of site-associated DNA sequencing (RADSeq) [131]. Several identified SNPs associated with plant diseases such as SNPs associated with anthracnose diseases in common bean [132], resistance to *Aphanomyces euteiches* in *Pisum sativum* [133], *Aphanomyces* root rot resistance against *Medicago truncatula* [134], resistance to *Uromyces pisi* in pea [135], verticillium wilt resistance in alfalfa [136], and resistance sites against *Plasmodiophora brassicae* in *B. napus* [137]. Using expressed sequence tag-based SNP markers, Kifuji et al. [138] mapped black rot resistance genes in cabbage and detected three QTLs. Similarly, Sharma et al. [139] developed a *Brassica carinata* F2 mapping population and mapped the black rot race 1 resistance locus Xca1bc. SNPs linked with plant colonization were found upstream of the Required for Arbuscule Development 1 (RAD1) locus, a positive regulator of arbuscular mycorrhizal (AM) fungal colonization in *M. truncatula* roots infected by *Phytophthora palmivora* [140]. Single nucleotide variant (SNV) is a substitution of a single nucleotide for another. Sometimes SNVs are known as SNPs, although SNVs and SNPs are not interchangeable. SNVs are only apparent in diploid or higher copy-number genomes and can be important for genomic differentiation for diploid/dikaryotic pathogenic fungi, as well as plants.

### 3.8. Chromosomal Polysomy or Length Polymorphism

Chromosomal polysomy occurs when an individual has at least one more chromosome than normal. Thus, instead of the expected two copies, there may be three or more copies of a chromosome. Core or dispensable chromosomes can become duplicated. Chromosomal polysomy occurs in various species, including plants, fungi, insects, and mammals [141]. Polysomy exists in many plant species, including *Brassica* species [142]. In plants, the mechanisms of polysomes includes non-disjunction (the failure of a pair of homologous chromosomes to separate), mis-segregation in diploids or polyploids, and mis-segregation from the multivalent interchange of heterozygotes [143]. In fungi, the polysomy of chromosome 13 was studied in yeast species *Saccharomyces cerevisiae* [144]. In addition, homologous chromosomes between individuals of the same species can have considerable length differences [51]. In fungi, chromosome translocations, deletion/insertion/duplication events, changes in repetitive DNA sequences, and dispensable chromosomes are the main causes of chromosome length polymorphisms [145]. In *Magnapothe grisea* and *F. oxysporum*, many families of TEs were discovered and linked as key factors affecting karyotypic instability [146]. 

### 3.9. Conditionally Dispensable Chromosomes

Unlike core chromosomes, conditionally dispensable (CDCs), or accessory chromosomes, are not essential for an organism. CDCs often differ from the core chromosomes in their size (typically less than 2.0 MB), gene content, and sequence characteristics [96]. Additionally, CDCs can be passed horizontally between isolates, potentially conferring new pathogenic characteristics on the recipient isolate [147]. In the case of plant pathogens, CDCs harbor virulence genes [51]. In fungi, CDCs were reported in several plant–pathogenic species, such as *Alternaria* species [148], *Fusarium solani* [149], and *F. oxysporum* [150]. Dispensable chromosomes were found in 14 species of fungi [151], including *Colletotrichum gloeosporioides* [152]. Plaumann et al. [153] showed that the deficiency of a dispensable chromosome in *Colletotrichum higginsianum* has critical effects on the fungus’ pathogenicity. Additionally, Ayukawa et al. [154] indicated that *F. oxysporum* f. sp. *conglutinans* (*Focn*) has multiple CDCs. The authors identified specific CDCs required for virulence on Arabidopsis, cabbage, and both. They also described a pair of effectors encoded on one of the CDCs required to suppress Arabidopsis-specific phytoalexin-based immunity. It was proposed genes playing a role in coding for host-specific toxins (HSTs), including AF-toxin from the strawberry pathotype [155], AK-toxin from the Japanese pear pathotype [156] and ACT-toxin from the tangerine pathotype [157], are positioned on CDCs. CDC loss can happen due to repeated sub-culturing, causing the fungus to shift from a pathogenic to saprophytic state [158].

### 3.10. De Novo or Orphan Genes 

De novo genes are species-specific (orphan) genes that derive from DNA sequences that previously lacked coding potential [51]. De novo genes are a subgroup of new genes that can code for proteins or serve as RNA genes [159]. De novo genes have different features than other genes in the genome. For example, de novo genes are shorter in size, have a lower expression rate, and contain more extensively varied sequences [160]. De novo gene birth is how new genes emerge from previously non-genic DNA sequences. De novo gene birth is essential for the divergence and adaptation of an organism [161]. The *BSC4* gene in *Saccharomyces cerevisiae* is an example of de novo gene birth [162]. The origins of de novo genes in plants have been widely studied [163,164,165,166]. Based on similarities to non-genic regions of *Arabidopsis lyrata*, almost half of the orphan genes in *A. thaliana* appear to have originated de novo [164]. Plant responses to the environment seem to be influenced by orphan genes [167]. For example, more than 80% of knockout mutants of unknown function genes in *A. thaliana* showed an altered phenotype when stressed, conferring either protection against, or serving as suppressors of, different abiotic stressors, notably oxidative and osmotic stresses [168]. A group of orphan genes was found in fungal pathogens limited to a single species or narrow clade. Pathogenic fungi may develop unique orphan genes to help infection or increase virulence. Because orphan genes lack homologs in closely related species, fungal effectors are ideal for orphan genes that developed for plant infection. Hundreds of orphan genes are encoded in the *Fusarium graminearum* genome [169]. The role of de novo or orphan genes in the pathogenic interactions and coevolution of pathogens with their host plants, however, remains unknown.

### 3.11. Epigenetic Modification of Gene Expression

Epigenetic modifications (e.g., DNA methylation, histone post-translational modifications, microRNAs, and the positioning of nucleosomes) are heritable alterations in gene expression patterns that occur without affecting the underlying DNA sequence and impacting the outcome of a locus or chromosome [170]. Epigenetic changes can affect only a particular gene (RNA interference (RNAi)-based silencing), or they can affect whole chromosomal regions (for example, epigenetic silencing of sub-telomeric regions due to histone modifications) [51]. Plant genomes are altered by various epigenetic pathways that regulate plant growth, development, and reproduction. Recent studies discovered many epigenetic factors participate in biotic and abiotic stress responses and adaptations in plants [171,172]. 

DNA methylation refers to adding a methyl (CH_3_) group to DNA and is an epigenetic mechanism that controls gene expression. As part of the plant’s defensive system, DNA methylation due to pathogen infection was reported in many plant species such as *Oryza sativa, A. thaliana, Nicotiana tabacum, Brassica rapa, Glycine max, Citrullus lanatus*, and *Aegilops tauschii* [173,174,175,176,177,178,179,180,181,182]. It was reported that pathogen detection provokes active changes in plant DNA methylation. For example, in *Arabidopsis*, infection with *P. syringae* pv. *tomato* DC3000 led to DNA hypomethylation in several genomic regions, such as peri/centromeric repeats and *Athila* retrotransposon [183]. Additionally, RNA-directed DNA methylation (RdDM) controls plant responses to pathogen attack. *Arabidopsis ago4* (ARGONAUTE 4, a vital component of the RdDM pathway) mutants feature reduced DNA methylation rates at different genomic locations and showed increased susceptibility to virulent *P. syringae* pv. *tomato* DC3000 [184]. Moreover, DNA demethylation in transposon-containing promoters enhances plant disease resistance. For instance, the *Arabidopsis ros1* (REPRESSOR OF SILENCING 1, a DNA demethylase) mutant presented greater susceptibility to *P. syringae* pv. *tomato* DC3000, which corresponded with substantially elevated cytosine methylation in a TE (*AtREP11*) present in the promoter of an *R* gene (*RMG1* or *At4g11170*) and consequently decreased gene expression [174].

As other epigenetic mechanisms, histone methylation and histone acetylation are active and reversible processes controlled by histone methyltransferases and histone demethylases and histone acetyltransferases and histone deacetylases, respectively [185]. Histone methylation and demethylation turn the genes in DNA “off” and “on”, respectively. Histone acetylation, on the other hand, is exclusively associated with gene activation [186]. In plant–biotic interactions, histone (de)methylation regulates plant defense. For example, the methyltransferases SDG8 and SDG25 were implicated in PTI, ETI, and systemic acquired resistance against bacterial and fungal pathogens. Moreover, *sdg8* and *sdg25* single and *sdg8 sdg25* double mutants displayed increased susceptibility to *B. cinerea* and *Pst* [187,188]. The role of histone (de)acetylation in plant–pathogen interactions on *Arabidopsis* has been examined in many studies [189,190,191]. In addition, the control of plant–pathogen interactions via histone (de)acetylation was investigated in the wheat histone acetyltransferase complex TaGCN5–TaADA2, which triggers wheat wax biosynthesis, thereby delivering wax signals for germinating conidia in fungal pathogen *Bgt* [192]. Additionally, rice HDAC OsHDT701 cooperates with the rice RNase P subunit Rpp30, and negatively controls rice defense responses to *M. oryzae* and Xoo by facilitating histone deacetylation at PRR and defense genes [193]. 

The transfer of ubiquitin to histone core proteins is known as histone ubiquitination. Histone ubiquitination, whether monoubiquitination or polyubiquitination, controls a series of cellular processes in plants. In *Arabidopsis*, histone H2B monoubiquitination (H2Bub) is carried out via HISTONE MONOUBIQUITINATION (HUB1) and HUB2 [194], which control *SNC1* and *RPP4* expression following *P. syringae* pv. *tomato* DC3000 attack [195]. 

### 3.12. Horizontal Gene/Chromosome Transfer 

The non-sexual transfer of genetic material, either a single gene or whole chromosomes between unicellular and/or multicellular organisms and acceptor organisms without a parent–offspring relationship is known as horizontal gene transfer (HGT). *Agrobacterium*-mediated transformation is the best example of HGT. After transferring a segment of *Agrobacterium* DNA into the host’s genome, *Agrobacterium* induces neoplastic growth or unregulated cell division, leading to crown galls or growing roots [196]. HGT plays an important role in the evolution of prokaryotic clones by providing new genes involved in pathogenicity and promoting adaptive traits [197]. Studies on fungal genomes suggest that HGT significantly influenced the evolution of pathogenic traits in fungal pathogens [198,199]. There is also evidence that some characteristics of fungal biology may allow for gene transfer. For example, the anastomosis of fungal conidia, germ tubes, and hyphae results in cytoplasmic cell–cell linkages between cells of different species [200]. In a previous study, Qiu et al. [201] analyzed genomic data from the fungal pathogen *Magnaporthiopsis incrustans*. The authors discovered two instances of exclusive sharing of HGT-derived gene markers between Magnaporthales and another lineage of plant–pathogenic fungi in the genus *Colletotrichum*. Yin et al. [202] identified 32 HGT events in *Valsa mali*, most of which were HGTs from bacteria, along with several others from eukaryotes. 

HCT between two vegetative incompatible biotypes of *C. gloeosporioides* [203] and the transfer of supernumerary chromosomes (extra chromosomes composed primarily of DNA not found in all representatives of the species) into nonpathogenic strains of *A. alternata* [204] are examples of HCT between fungi. Moreover, the horizontal transfer of chromosome 14 from *F.* *oxysporum* f.sp. *lycopersici* to nonpathogenic *F. oxysporum* strains confers the pathogenicity of these strains towards tomato [64].

### 3.13. Hybridization

The process of interbreeding individuals of different varieties or species to produce a hybrid is called hybridization. Breeding programs have yielded extensive hybridization between individuals of the same or different plant species. The introgression of genes for disease resistance between species has been widely studied in *Brassica* species. For example, chromosome B4 from *Brassica nigra* was introgressed into the rapeseed variety “Darmor” as a source of resistance against *L. maculans* (causal agent of blackleg) and led to high resistance [205]. Similarly, a B genome chromosome was introgressed from *B. carinata* to *B. napus* indicating high resistance against *L. maculans* [206].

Other cases of resistance transfer through hybridization include hybridization between *B. carinata* (donor) and *B. oleracea* to enhance resistance against *Erysiphe polygoni* (which can cause powdery mildew disease) [207], the transfer of black rot resistance from *B. carinata* to *B. oleracea* [208], the transfer of brassica leaf blight resistance (caused by *Alternaria brassicae*) from *B. hirta* to *B. juncea* [209], and the production of powdery mildew resistance from *B. carinata* to *B. oleracea* through embryo rescue followed by backcrossing to *B. oleracea* [207]. From the pathogen side, Bertier et al. [210] showed that hybridization increased *Phytophthora* clade 8b pathogenicity. 

### 3.14. Polyploidization

Polyploidization, or whole-genome duplication, refers to the acquisition of extra sets of chromosomes in a cell or organism and frequently occurs in vascular plants. Polyploidization is an essential aspect of plant evolution and can significantly modify a plant’s genetic make-up, physiology, morphology, and ecology within one or more generations [211]. Polyploidization can affect biotic interactions and resistance to pathogens, with polyploids generally having enhanced pathogen resistance. Differences between diploids and polyploids in *R* genes reflects altered pathogen resistance [212]. For example, polyploidy can increase resistance within the gene-for-gene interactions that underlie many host–pathogen interactions and where genotype × genotype interactions are important [213]. Quantitative resistance against *P. infestans* and *Tecia solanivora* in 4x potato was, moreover, observed using QTL analysis [214]. In a previous study, neopolyploids of a monogenic resistant apple cultivar showed increased resistance to *V. inaequalis* compared to diploid cultivars [215]. Another study found that synthetic tetraploids of Livingstone potato (*Plectranthus esculentus*) were more resistant to root-knot nematodes than diploids [216]. Pathogens can also change ploidy during infections; this phenomenon occurred with *P. infestans*, which caused the Great Irish Potato Famine [217]. From the evidence available, polyploidy can induce changes in pathogen interactions and increase disease resistance by regulating genome expression, resulting in alterations in physiological characteristics, hormone biosynthesis, and improved antioxidant systems [218], which make polyploids better competitors than diploids. For example, polyploidy was investigated in *Bremia lactucae* by Fletcher et al. [219] who reported a high incidence of heterokaryosis in *B. lactucae*. Heterokaryosis has phenotypic consequences on fitness that may include an increased sporulation rate and qualitative differences in virulence. 

## 4. Conclusions and Perspectives 

As selective agents, pathogens play a crucial role in plant evolution. However, this role depends on the extent of genetic variation among resistance traits and their relationship with host robustness. Deciphering plant and pathogen genome content alongside the evolutionary relationships of ancestral species and their descendants can be beneficial in developing resistant varieties. Although our understanding of plant–pathogen interactions has advanced considerably in recent decades, there are still many questions regarding the role of genetic variation and mutational events in evolution and plant–pathogen interactions; for example, what are the key factors that influence genetic variation? How do genetic variation and mutational events lead to disease resistance? What form of genetic variation promotes disease resistance, and how does genetic variation add to breeding resistance and the development of pathogen-resistant crops for human food sustainability? To address these questions and to further advance our knowledge of plant–pathogen interactions and disease management, biomolecular and genomic research tools such as next-generation sequencing technology and functional genomics, various ‘omics’ technologies, and databases for metabolic modeling are essential [220]. Omics tools involving genomics, proteomics, transcriptomics, and metabolomics approaches, along with bioinformatics methods, have spurred the growth of our knowledge on plant–pathogen interactions to a large extent and continue to play a major role in identifying QTL/candidate *R*/pathogenicity genes to genetically improve crop species that are resistant to pathogens. In addition, genome editing, which is one of the most important biotechnological tools, has increased our biological knowledge and lead to rapid progress in agriculture and crop breeding. Furthermore, combined with pangenomics, genome editing facilitates functional and comparative analyses. Finally, we expect genomic variation to create a paradigm shift in resistance breeding and to help crop breeding achieve accelerated crop improvements to contribute to a food-secure world. In this context, efficient crop breeding programs and recent advances in genotyping and phenotyping will accelerate crop breeding and pave the way toward developing the next generation of disease resilient and high-performance crop varieties.

## Data Availability

The study did not report any data.
